# Do physiotherapists have a role to play in the Sustainable Development Goals? A qualitative exploration

**DOI:** 10.4102/sajp.v75i1.466

**Published:** 2019-04-25

**Authors:** Sholena Narain, Desmond Mathye

**Affiliations:** 1 Department of Physiotherapy, Sefako Makgatho Health Sciences University, Pretoria, South Africa

**Keywords:** physiotherapy, sustainable development goals, maternal health, child mortality, HIV and AIDS, inclusive primary education, chronic diseases of lifestyle

## Abstract

**Background:**

While physiotherapists appear to be ideally positioned as key role players in achieving the health- and education–related Sustainable Development Goals (SDGs), few studies have examined the complete scope of physiotherapy practice in addressing the SDGS. Considering the broad scope of physiotherapy practice, physiotherapists are a valuable resource that the South African government can utilise to address their workforce shortages in achieving inclusive primary education, promoting gender equality, reducing child mortality, improving maternal health, and combating HIV and AIDS, and other diseases.

**Objectives:**

The aim of this study was to understand the roles of physiotherapists in the SDGs.

**Method:**

A qualitative, exploratory and descriptive approach was used. Semi-structured telephonic and Skype interviews were utilised to collect data from nine physiotherapists with PhDs working in academic institutions. Data were transcribed verbatim by the first author and verified by the second author. Data were entered into NVivo^®^ Version 10. An inductive approach to qualitative data analysis was used. *In vivo* and open coding was used to generate codes and themes.

**Results:**

The following roles were highlighted: (1) address HIV and AIDS, tuberculosis and other chronic diseases of lifestyle; (2) improve maternal health; (3) reduce child mortality; (4) empower women and (5) achieve inclusive education for children, especially children with disabilities.

**Conclusions:**

Physiotherapists are well suited to address the SDGs of promoting gender equality and empowering women, reducing child mortality rates, improving maternal health, achieving inclusive primary education and combating HIV and AIDS, tuberculosis and other chronic diseases of lifestyle. Physiotherapists have a valuable role in addressing the quadruple burden of disease in South Africa and assisting the government with the current health resource crisis.

**Clinical implications:**

The results of this study will assist to move patient management from a more curative approach to health promotion and prevention. In addition, this study highlights the valuable role of physiotherapists in assisting and supporting the development agenda for ‘Transforming our world: the 2030 Agenda for Sustainable Development’.

## Introduction

**FIGURE 1:**
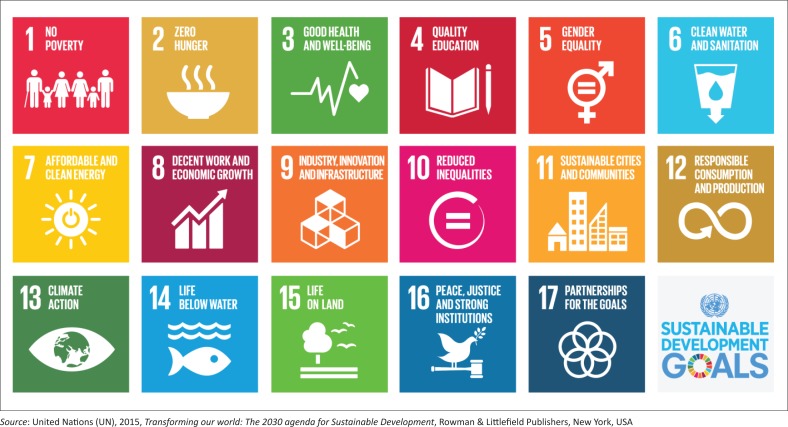
The 17 sustainable development goals.

In the 21st century, the physiotherapy profession in South Africa needs to address societal needs and the global burden of disease. The Millennium Development Goals (MDGs) were adopted by world leaders in September 2000 at the Millennium Summit (United Nations 2000). The MDGs were aimed at eradicating poverty and hunger, achieving universal primary education, promoting gender equality, reducing child mortality, improving maternal health, combating HIV and AIDS, malaria and other diseases and ensuring a sustainable environment (United Nations 2000). As the MDGs approached their deadline, a bold new agenda emerged, the Sustainable Development Goals (SDGs). On 25 September 2015, the 194 countries of the United Nations General Assembly adopted the 2030 Development Agenda titled ‘Transforming our world: The 2030 agenda for Sustainable Development’ consisting of 17 goals (United Nations 2015). The 17 goals are illustrated in Figure 1.

According to the World Health Organization (WHO) (2016), health is a contributor to several of the SDGs, but health priorities of the post-2015 agenda for sustainable development will remain aspirational unless strategies to transform the health workforce capability are put in place. Physiotherapists are an ideal health workforce to address the good health and well-being, quality education and gender equality goals.

### Good health and well-being

Physiotherapy focuses on management, health promotion and prevention of a broad range of lifestyle conditions (Dean 2009). With an estimated 35 million people living with HIV (UNAIDS 2011), HIV remains a major global health catastrophe. The effectiveness of physiotherapy in the management of persons living with HIV and AIDS has been well documented (Grace & Combrink 2015; Myezwa & Stewart 2012; Pullen 2017).

Exercise, in particular as part of behaviour modification and improvement in lifestyle, has been shown to contribute positively to preventing complications of HIV and increasing longevity in persons living with HIV (O’Brien et al. 2016). In addition, in South Africa, tuberculosis (TB) is the most commonly reported cause of death in adults (Abdool Karim et al. 2009), and the economic burden on the household in South Africa could potentially be severe with an estimated 530 000 people being infected with TB per annum (Creswell et al. 2014). Exercise prescription forms the basis of a management programme for patients with chronic respiratory disease (Dyer, Singh & Stockley 2002).

The Global Burden of Disease Study performed in 2010 reported that there are more young adults suffering from non-communicable diseases (NCDs) that could lead to death and disability worldwide (Horton 2012). In addition, the cost of amputations, artificial limbs, wheelchairs and cardiac surgery place an enormous financial burden on health budgets (Lawrence 2011). Physiotherapists play a significant role in combating the chronic diseases of lifestyle, such as hypertension, which is responsible for one in every eight deaths worldwide (Punia et al. 2016) and an essential role in the multidisciplinary approach of obesity (Alexander, Rosenthal & Evans 2012).

Physiotherapists have a valuable role in improving maternal health, and several studies have indicated the benefits of regular exercise in pregnancy that will effect an improvement in physical fitness and cardiovascular endurance (Wolfe & Davies 2003); prevention of excessive weight gain and glucose intolerance (Mottola & Ruchat 2011); training and strengthening of the muscles necessary for facilitating labour (Riberio & Milanez 2011) and improvement in psychological adjustment to changes in pregnancy (Wolfe & Davies 2003). Furthermore, exercise in pregnancy is associated with a decrease in many common problems of pregnancy (Wadsworth 2007).

Infant and child mortality forms part of South Africa’s quadruple burden of disease (DOH 2011). Physiotherapists have an integral role to play in decreasing child mortality. Balabanova (2013) reported that improved child health could be achieved with relatively few resources if these are used strategically. According to Makhabah, Martino and Ambrosino (2013), postoperative pulmonary complications are one of the leading causes of prolonged hospital stay, increased health care expenses, morbidity and mortality. The authors indicated that post-surgical physiotherapy for children in intensive care units decreases the risks of postoperative pulmonary complications. In addition, physiotherapy programmes provide innovative and cost-effective methods of decreasing complications and patient’s ventilator dependency (Balabanova 2013).

### Quality education

Children with disabilities are less likely to start school and tend to have poor prospects of remaining and being promoted in school (Filmer 2008). Physiotherapists play an integral role in addressing quality education for children with disabilities. To achieve primary education in children with disabilities, investment in school infrastructure and staff needs to be augmented to ensure that children with disabilities obtain special education and additional support during their schooling education (WHO 2011).

### Gender equality

Women empowerment is an important issue in today’s world, as women and girls remain victims of gender inequality (Read & Gorman 2010). In many countries, women and girls have less access to education, which is an essential predictor of well-being (Bobbitt-Zeher 2007). The various methods for empowering women include economic independence (Ahmed et al. 2010), enhancement of skills (Sharf 1997) and promotion of health knowledge (Porr, Drummond & Richter 2006). Physiotherapists are well positioned to empower women and girls towards acquiring and understanding basic health information and consequently positively influencing their judgements and attitudes towards health behaviours (Harrison, Mackert & Watkins 2010).

Although physiotherapists appear to be ideally positioned as key role players in achieving the health and well-being as outlined in the SDGs, few studies have examined their role in addressing the education and gender equality goals. The purpose of this study was to describe the opinions of expert academic physiotherapists on the role of physiotherapists in the SDGs.

## Methodology

To understand the role of physiotherapists in the SDGs, a qualitative, exploratory and descriptive study was undertaken. The study sample consisted of nine physiotherapists, registered with the Health Professions Council of South Africa (HPCSA), who were holders of a PhD degree. All the participants were employed at South African universities and occupied various academic ranks. As a result of their qualifications and experience, the nine participants are referred to as ‘expert’ physiotherapists. The demographic characteristics of all participants are presented in Table 1. Three participants were from the University of Cape Town, one from the University of KwaZulu-Natal, one from the University of the Witwatersrand, one from Stellenbosch University, one from North-West University and two from the University of Limpopo. The North-West University and the University of Limpopo do not offer degrees in physiotherapy; however, the two participants from these universities were actively involved in physiotherapy-related research.

**TABLE 1:** Demographic characteristics of participants.

Code	Age	Gender	Rank	Qualifications	Number of publications	Experience (years)
Exp 1	42	Male	Associate Professor	BSc Physiotherapy, MPH, PhD	11	17
Exp 2	42	Female	Associate Professor	BSc Physiotherapy, PGD (Ethics), PhD	63	15
Exp 3	36	Female	Senior Lecturer	BSc Physiotherapy, MSc Physiotherapy, PhD	29	9
Exp 4	55	Male	Professor	BSc Physiotherapy, LLB, MSc, PhD	44	18
Exp 5	37	Female	Lecturer	Bachelor of Physiotherapy, MSc Physiotherapy, PhD	15	10
Exp 6	48	Female	Professor	Bachelor of Physiotherapy, MSc Physiotherapy, PhD	28	20
Exp 7	64	Female	Professor	BSc Physiotherapy, MPhil Physiotherapy, PhD	94	36
Exp 8	55	Female	Postdoctoral Fellow	BSc Physiotherapy, MMedSc Rehabilitation, PhD	17	12
Exp 9	44	Female	Associate Professor	BSc Physiotherapy, MPH, PhD	17	15

The first author conducted semi-structured interviews using guiding questions and demographic forms to collect data. The guiding questions included the following: (1) what role do physiotherapists play in the public sector? (2) what role do physiotherapists play in the private sector? and (3) what is the anticipated role of physiotherapy in the national health insurance (NHI)? Field notes were used as a supplementary data collection tool. The nine participants, known to the researchers, were invited to participate via email together with an invitation leaflet, demographic information sheet and informed consent form. Upon receipt of their informed consent to participate, the participants were contacted again via email to set up a suitable time for the interviews. Participants were allowed to choose between a telephonic and a Skype^®^ interview. Eight participants chose telephonic and one chose Skype. All interviews were conducted in English and recorded using a digital audio recorder.

### Data analysis

Data collected were analysed using a deductive approach. Preliminary analysis of the data began during the interviews. A verbatim transcription of the audiotaped data was done and converted into a Microsoft Word^®^ document. The transcripts were verified by the first author for accuracy and uploaded into NVivo^®^ Version 10 for coding.

### Trustworthiness

According to Guba and Lincoln (1981), credibility, dependability, confirmability and transferability are the four criteria used to demonstrate trustworthiness of a qualitative research. To ensure credibility, the authors used a well-established methodology, peer review and existing literature to gauge the degree to which the research results were congruent with the results from latest research. Dependability was ensured by a detailed process of data collection and analysis. An audit trail of data collection and analysis was used to ensure confirmability. Transferability was ensured by a thorough presentation of the methodology to allow the reader to establish the significance of this study relative to studies in a similar context.

### Ethical considerations

Ethical approval was obtained from the Sefako Makgatho Health Sciences University’s Ethics Committee (reference number: SMUREC/H/178/2016: PG). All participants signed an informed consent form, wherein anonymity, confidentiality, right to withdraw, non-maleficence and avoidance of deceptive practices were guaranteed (Guraya, London & Guraya 2014).

## Results

Participants in this study identified three SDGs that physiotherapists play a role in (1) good health and well-being, (2) quality education and (3) empowering women.

### Theme 1: Good health and well-being

Participants believed that physiotherapists have a major role to play in South Africa’s quadruple burden of disease and specifically highlighted the integral role in HIV/AIDS. Participants felt that physiotherapists should be key role players in managing the disease and its complications, and also play a major role in addressing the health promotion, education and prevention of the disease. Participants expressed that physiotherapists have a role in screening, educating and preventing the complications of the disease. The experts were of the opinion that physiotherapists should play a greater health promotion and prevention role in HIV/AIDS and proposed that the undergraduate physiotherapy curriculum needs to include health promotion and prevention training in HIV (see Table 2).

**TABLE 2:** Theme 1 – Role in good health and well-being – Part 1.

Category	Code	Quotation
HIV/AIDS	Promotion and prevention	‘Physios [*sic*] have multiple roles in people living with HIV … going into communities and teaching about the importance of endurance to ARTs, maintaining diet, exercise, talk about sexual behaviour’. (Exp 5)
	Exercise prescription	‘With HIV/AIDS, people have reduced exercise tolerance; they could benefit from health promotion, exercise, health counselling and testing services. That is definitely a role that physiotherapists play’. (Exp 3)
	Educate and empower	‘Physiotherapists are well suited to do health education with HIV patients to prevent the spread of the disease. Physiotherapists have a significant role in empowering these young people in the importance of their safety and being responsible for their behaviour’. (Exp 6)
	Palliative care	‘Physio [*sic*] is really important in the palliative care context and has been shown to improve quality of life in terminally ill and end of life care by optimising independent function’. (Exp 2) ‘Our role in palliative or end-stage HIV-patients is making the patient as comfortable as possible’. (Exp 5)
Tuberculosis	Screening and prevention	‘Quick tests like peak flow, and spirometry that can be done in clinics to identify lung pathology in patients’. (Exp 3) ‘There’s nothing stopping physios [*sic*] from going into communities and talking about TB’. (Exp 5) ‘Physios [*sic*] prevent complications of chronic disease’. (Exp 2)
Chronic diseases of lifestyle	Hypertension	‘Hypertension is one of the most common conditions seen in primary health care; we educate them on the consequences of high blood pressure’. (Exp 1) ‘Physios [*sic*] optimise general health, for example, smoking cessation policies’. (Exp 2) ‘We are already involved in primary healthcare clinics with exercise programmes to minimise the effects of chronic diseases of lifestyle’. (Exp 7) ‘We play a preventative role in the adolescent population at risk for high blood pressure’. (Exp 4)
	Diabetes	‘Diabetes is a burden in South Africa. Physios [*sic*] are involved in the prevention in developing programmes on eating healthy, regular exercise and speaking to children and schools about the importance of exercise’. (Exp 5) ‘Our role is to educate diabetic patients at clinics, about diabetes and how exercise can assist in alleviating some of the symptoms’. (Exp 1)
	Obesity	‘Remember our duty is to fight obesity, and who better placed to fight obesity than a physiotherapist. Obesity is a risk factor for all chronic diseases: hypertension, diabetes, hypercholesteremia. We play a significant role in ensuring that our children are eating healthy so that they do not progress to being obese’. (Exp 6) ‘In schools, we do fitness testing, as markers of disease or risk factors for NCDs. In adolescents we know poor physical fitness is a predictor of future non-communicable diseases’. (Exp 3)

NCD, non-communicable disease; ART, anti-retroviral therapy.

In addition, participants felt that physiotherapists should play a major role in the prevention, education and management of NCDs by addressing issues of obesity, healthy eating, exercise and smoking cessation. Some participants believed that physiotherapists should extend the health education and prevention role to those who are not sick but may be at risk for chronic diseases of lifestyle. The experts were of the opinion that physiotherapists have a role in the management of diabetes and obesity by introducing preventative health programmes, directly fighting obesity and alleviating the symptoms of diabetes, as depicted in Table 2. The participants were also of the opinion that physiotherapists have an essential role to play in improving maternal health and reducing child mortality (see Table 3).

**TABLE 3:** Theme 1 – Role in good health and well-being – Part 2.

Category	Code	Quotation
Improving maternal health	Health promotion messages	‘Physiotherapists have a very active and important role to play in reproductive health and maternal health services … Working in close collaboration with nursing staff at antenatal clinics … health promotion messages … preventing a lot of developmental disabilities’. (Exp 3)
	Complications of delivery	‘Physios [*sic*] play a role in women’s health by preventing complications of delivery’. (Exp 2)
	Breastfeeding advice	‘They can go into clinics and talk about the importance of breastfeeding’. (Exp 5)
Reducing child mortality	Promote healthy behaviours	‘Paediatric and child health services can definitely include physiotherapists … the link between women’s maternal health and child health is an important role that physiotherapist play … Encouraging pregnant women to eat well, be active, not engage in any harmful behaviours such as: taking alcohol, smoking, drugs etc. … all those link to foetal alcohol syndrome’. (Exp 3)
	Reduce mortality rates	‘What role can physiotherapy play in maternal and child health? A huge one!… We should all get involved in child and maternal health, antenatal care … postnatal care … we have to cap down the high incidence or rates of infants and mother mortality rates’. (Exp 1)

### Theme 2: Empower women

The majority of physiotherapists in South Africa are females, and the study found that the participants believed that physiotherapists could play a significant role in empowering older women to mentor younger women in communities and to also empower women to become more actively involved in parenting, home care and growing and preparing their own food (see Table 4).

**TABLE 4:** Theme 2 – Empower women.

Code	Quotation
Growing own food	‘We can also play quite a big role in terms of empowerment … Elderly women who really can step up and take a leadership role…you have so much to give to the younger women; you have so much to show them on how to run their houses … school and stimulate their children … To empower that resource… that I think is the role of the physiotherapists’. (Exp 8)
Mentor young women	‘The other messages that you can instil on these young girls can make these girls better parents, better adults, when the time comes … that is where you can play a significant role’. (Exp 6)

### Theme 3: Achieve inclusive education for children with disabilities

Participants were of the view that physiotherapists play a major role in achieving inclusive education for children, especially children with disabilities by early screening and referral, optimising their function in the school environment and working on an inter-sectoral platform with the school education staff. They have a role in promoting and providing health promotion and education training regarding children with disabilities (see Table 5).

**TABLE 5:** Theme 3 – Achieve inclusive education for children with disabilities.

Code	Quotation
Optimise function	‘In special schools, there are very specific roles in terms of providing therapy to children with chronic disabilities … both within the classroom and therapy based specifically so optimising their function in the school system’. (Exp 2)
Screening and referral	‘Design health promotion programmes, increase physical activity levels … to screen and identify learners who may have developmental problems…playing an important role in ensuring that learners who do have specific health conditions are identified early, screened and then referred appropriately’. (Exp 3)
Educate school staff	‘Within our scope of practice so we can even talk to educators about handling children with disabilities’. (Exp 5)

## Discussion

This study highlights the integral role of physiotherapists in addressing three major goals of the SDGs, namely achieving good health and well-being, empowering women and achieving inclusive education for children with disabilities. South Africa’s quadruple burden of disease consisting of HIV and AIDS, TB, high maternal infectious diseases and undernutrition resulting in neonatal and child mortality has led to an increase in the burden of chronic NCDs leading to disability and early death (Schaay, Sanders & Kruger 2011).

### HIVand AIDS and tuberculosis

The crucial role of physiotherapists in the function and well-being of people living with HIV (PLHIV) is often not acknowledged (Malan 2012), and the urgent need for therapy resources in the area of HIV management especially in the motor and cognitive development challenges facing HIV-infected children has been neglected and underscored (Potterton et al. 2010). As such physiotherapists working in all sectors need to respond proactively and energetically to the HIV epidemic to improve the relevance and the value of the profession (Myezwa & Stewart 2012).

Cobbing et al. (2013) agree that physiotherapists should be key role players in providing rehabilitation and adding value to the lives of PLHIV. However, to promote physiotherapy’s critical role in HIV and improve the standing of the profession, the authors recommend that the role be promoted with improved training, research, proactive clinical intervention and collaboration with other health professionals.

According to Myezwa (2008), the collective curriculum at the eight schools of physiotherapy in South Africa had integrated HIV into some of the physiotherapy specialisation areas; however, gaps were found in the physiotherapist’s role in HIV prevention treatment and care. The author further noted that this gap might restrict the physiotherapists’ role in health promotion and secondary and tertiary prevention of problems around HIV and AIDS.

Considering that 7.1 million South Africans are estimated to be infected with HIV and AIDS (Ngcobo 2017), and the disease may very well be the biggest underestimated threat to future generations, the physiotherapy profession cannot continue with business as usual. Instead, physiotherapists need to ensure that extraordinary measures are taken to address the health promotion and prevention aspect of HIV and AIDS.

It is estimated that by 2020 approximately 1 billion people will be newly infected with TB and 35 million people will die unless the fight against TB is strengthened (WHO 2012). Considering that in South Africa, TB is the most commonly reported cause of death in adults, and most TB deaths are associated with HIV infection (Abdool Karim et al. 2009), it would appear necessary for physiotherapists to increase their health promotion and prevention roles in HIV and AIDS.

### Chronic diseases of lifestyle, the non-communicable diseases and obesity

According to WHO (2013a), the four main NCDs leading to the highest incidences of mortality are (1) cardiovascular disease, (2) chronic respiratory illness, (3) diabetes and (4) cancer, and these diseases share four risk factors: tobacco use, physical inactivity, harmful alcohol use and unhealthy diets, which lead to raised blood pressure, obesity, hyperglycemia and hyperlipidemia. There are approximately 3.2 million deaths annually that are attributed to insufficient physical activity alone (WHO 2013a).

The burden of NCDs has been described by the United Nations Secretary-General Ban Ki-moon as ‘a public health emergency in slow motion’ (UN 2000:2173). Although chronic diseases in both low and high resource countries are a major risk to the global economy, all NCDs can either be prevented or, if identified early, treated and managed in a way that significantly reduces disability, financial and societal costs, and prolongs healthy years of life (World Health Professions Alliance 2011).

In light of this, it is essential for physiotherapists to re-evaluate their role in the prevention and treatment of NCDs (Van Rooijen & Van der Spuy 2000) as physiotherapists are equipped with the knowledge to understand the initial cause of ailments and can, therefore, be leaders in prevention by offering advice and guidance on how to avoid the root causes of disability (Metz et al. 2012). Although physiotherapists have the potential to address important risk factors associated with NCDs and not just physical activity levels, physiotherapists cite insufficient time and inadequate competencies to do so, recommending that practice standards and educational requirements need to be addressed (O’Donoghue et al. 2014).

Although this study found that physiotherapists are well placed to deal with the issue of obesity, You et al. (2012) reported that physiotherapists are unclear about their roles and scope of practice in the management of obesity which has inhibited the role they play. The Chartered Society of Physiotherapy (2015) stated that if physiotherapists could use every opportunity to assess and advise on all four risk factors in every patient encounter, then the physiotherapy profession would be making a significant contribution to combating the burden of NCDs.

### Role of physiotherapy in improving maternal health and reducing child mortality

South Africa has unacceptably high levels of maternal mortality, and the number of women and girls who are dying during pregnancy or shortly after giving birth has increased dramatically since 2000 (NDOH 2012). In 2012, the maternal mortality rate was at 269 deaths per 100 000 live births of which experts suggest 60% of maternal deaths in South Africa are avoidable. Pattinson (2005) concurs with findings of our study that proper and timely antenatal care plays a significant role in improving maternal and child health and preventing maternal deaths.

The WHO (2016b) recommends a minimum of four antenatal care visits, which serve as an opportunity to provide vital health information to women and girls relating to lifestyle risks and to offer social support and counselling. Although Sajan (2013) indicated the need to increase awareness about physiotherapy interventions among pregnant females and professionals in the antenatal clinics, the responsibility of this awareness rests with the physiotherapy profession. Physiotherapy can contribute significantly to women’s health by promoting awareness through campaigns, health education and interaction with other health professionals (Sajan 2013).

Considering the globally growing shortage of 7.2 million health care workers, and around 90% of all maternal deaths and 80% of stillbirths occur in countries that lack trained health care workforces (WHO 2013b), utilising physiotherapy resources in addressing South Africa’s quadruple burden of disease will have benefits to both patients and national health. However, according to Odunaiya et al. (2013), physiotherapy is underutilised because of a lack of understanding of what physiotherapists can offer in maternal and child health. The authors recommend collaboration between physiotherapists, obstetricians and gynaecologists and a national initiative to market and promote the role of physiotherapy in women and child health.

Improved maternal health is directly linked to decreased infant mortality and participants in this study believed that physiotherapists should not view infant mortality as a field to be championed by paediatricians, but instead be key team members in the multi-professional team to ensure decreased child mortality in South Africa.

Congenital heart disease affects about 8–10 children per 1000 live births (Pinto et al. 2004), and heart surgery leads to a number of respiratory complications such as atelectasis, pneumonia, pleural effusion, pneumothorax, chylothorax, pulmonary hypertension, pulmonary hemorrhage and diaphragmatic paralysis (Felcar et al. 2008). Physiotherapy in the pre- and the postoperative period is indicated in paediatric cardiac surgery to reduce the risk of pulmonary complications and to contribute to the appropriate ventilation and successful extubation of paediatric patients (Nicolau & Falcão 2010).

Childhood diseases may lead to long-term impairments in gross and fine motor skills, loss of flexibility, behavioural problems and cognitive impairment such as lower attention spans, language impairment, learning disabilities and low IQ scores (Waber et al. 2014). However, physical activity and exercise prescription in children with neuromotor disabilities are seldom practised in resource-limited settings (Bekele & Janakiraman 2016).

### Role in empowering women

Women empowerment is an important subject of today’s world as women and girls remain victims of gender inequality (Read & Gorman 2010). In many countries, women and girls have less access to education, which is an essential predictor of well-being (Bobbitt-Zeher 2007). When addressing women’s lives, it is important to examine the underlying social, cultural, environmental, epidemiological and economic determinants of health (Marmot et al. 2008).

Women and girls have specific health needs, and health systems around the world seem to be failing them, and the highest mortality and disability rates are found in Africa (WHO 2009). Considering that the majority of South African physiotherapists are females who play a number of roles in South African society, such as academics, researchers, clinicians, managers and pioneers in many other areas of their lives, they are well positioned to be role models in South African society and empower girls and young women to excel in all areas of their lives and not only health care. The physiotherapy profession should reconsider a model that includes the social, political and economic contexts of women empowerment in South Africa (Marmot 2005).

### Role of physiotherapy in achieving quality education for children, especially children with disabilities

In post-apartheid South Africa, children with disabilities face several barriers in the education system (ACPF 2011) which has resulted in the substantial exclusion of children with disabilities from education (Department of Education 2001). In general, children with disabilities are less likely to start school and have lower rates of staying and being promoted in school (Filmer 2008).

Although the WHO (2011) indicates that physiotherapists are well positioned to ensure that primary education is achieved for children with disabilities, findings of our study show that physiotherapy services are inaccessible to children in poor communities. It is essential for the physiotherapy profession to ensure improved access to physiotherapy services to schools in under-resourced areas, advocate for increased investment in school infrastructure and personnel so that children with disabilities that are identified as having special educational needs obtain the needed support and continue to receive that support during their education (WHO 2011). In addition, the availability of speech and language therapy, occupational therapy and physiotherapy to learners with moderate or significant disabilities would promote inclusive education and ensure that primary education is achieved (WHO 2011).

Mukhopadhyay, Nenty and Abosi (2012) reported that the implementation of inclusive education for learners with disabilities is a complex process and requires the active involvement of all stakeholders and the positive interaction between multiple systems to be successful. For example, Struthers (2005) found a gap between the policy of an inclusive education system for South Africa and the implementation of the policy. The author further noted that school-based therapists’ support to schools would be most effective by working together with other members of the district-based support team, within a health promoting schools framework. However, to achieve this goal, therapists need to have appropriate competencies to offer the most effective support to the teachers, parents and the education system, and competencies for the provision of direct learner support (Struthers 2005).

Sonday (2012) agrees that collaboration between all role players in the system at all levels is vital to address the challenges of implementation and for inclusive education to become a reality. In addition to collaboration and developing the appropriate competencies, physiotherapists will need to address the barriers to education in the country. In South Africa, the social conditions, such as poverty, unemployment, illiteracy among parents, violence, teenage pregnancy and drug abuse, need to be addressed for successful education to take place (Chisholm 2005).

Currently, in South Africa, physiotherapists are not included in school-based teams; embedding physiotherapists in the school-based framework will provide valuable support and assist learners in the development of skills, such as the motor function skills required to be integrated into the curriculum. Apart from the direct support to learners, physiotherapists’ indirect support to teachers, other therapists, parents and community members will ensure that barriers to inclusive education are addressed. In addition, the role of physiotherapy in achieving inclusive education promotes the constitutional values of equality, freedom from discrimination and the right to a basic education for all learners, especially those who experience barriers to learning.

The highly skilled participants in our study were all physiotherapists and all had PhD degrees. However, this does not necessarily mean that they had expert knowledge with regard to all of the issues that were included in the interviews, but all had expertise in many of the issues under discussion.

## Conclusion

It is evident from the results of our study that physiotherapists are well suited to address the SDGs of health and well-being, achieving quality education for children, especially children with disabilities and empowering women. Our study reflects the valuable role that physiotherapists play in assisting and supporting the development agenda for ‘Transforming our world: The 2030 agenda for Sustainable Development’. There is limited literature on the role of physiotherapy in the SDGs. Further research is necessary to investigate the complexities of the SDGs and physiotherapy’s complete scope of practice in the SDGs.
